# Perspective: can interfacial defects enhance graphene’s reactivity for gas–surface reactions under ultra-high vacuum?

**DOI:** 10.3389/fchem.2026.1850809

**Published:** 2026-06-12

**Authors:** Abdolvahab Seif, Thomas Stach, Trung T. Pham, Md Arif Uddin, Jean-François Colomer, Robert Sporken, Alberto Ambrosetti, Pier Luigi Silvestrelli, Uwe Burghaus

**Affiliations:** 1 Dipartimento di Fisica e Astronomia, Università di Padova, Padova, Italy; 2 Department of Chemistry, University of Turin, Turin, Italy; 3 Department of Chemistry and Biochemistry, North Dakota State University (NDSU), Fargo, ND, United States; 4 Department of Physics, Namur Institute of Structured Matter (NISM), University of Namur, Namur, Belgium

**Keywords:** catalysis, density functional theory, graphene, kinetics, spectroscopy

## Abstract

A very recent experimental study [*Proc. Natl. Acad. Sci. U.S.A. 123 (10) e2524790123*] demonstrated that non-functionalized graphene (Gr) supported on silicon(111) catalyzed the decomposition of small sulfur-containing compounds under ultra-high vacuum. Accompanying density functional theory (DFT) calculations identify grain boundary defects (GBDs) and interfacial defects as the active sites. Functional carbon catalysts still encounter skepticism within the catalysis and surface science communities.

## Introduction

Despite numerous successful examples in the literature for solid–liquid phase reactions (Borisova et al., 2013; Esrafili et al., 2016; Faye et al., 2016; Komeily-Nia et al., 2020; Long et al., 2011; Mohammadi et al., 2017; Su and Loh, 2013; Widjaja et al., 2017) catalyzed by graphene (usually flakes), we often encounter substantial criticism at scientific meetings and in U.S. proposal review panels stating that graphene (Gr) could not act as a catalyst. This skepticism is usually rooted in the fact that free-standing, unsupported graphene is generally non-reactive. However, often overlooked is that graphene’s reactivity can be “defect- and support-driven,” as correctly stated in the title of this volume. In principle, graphene’s reactivity can be engineered and customized toward specific applications. Specifically, the skepticism expressed by some colleagues in the catalysis, surface science, and materials science fields often concerns the following.

1. The lack of d-electrons in graphene, which are considered important for enhancing reactivity in conventional metal-based catalysts.
2. The apparent lack of evidence that the observed reactions are catalytic rather than stoichiometric, which would preclude the formation of a catalytic cycle (adsorption of reactants→desorption of products→ads …).
3. The apparent lack of evidence ruling out intercalation effects, implying that the reactivity originates from the support rather than from graphene itself (i.e., the reactions proceed underneath the graphene sheet on the support).
4. The apparent lack of evidence ruling out large vacancy defects in graphene, again suggesting that the observed reactivity is related to the support.

If all these criticisms are disproven, the most common statement made by critics is as follows.

5. “There are likely some unidentified impurities in the graphene” and that render it reactive (citing a very recent referee report we received on a study). Similarly, “some kind of unknown experimental artifact (/mistake) affected the results.”

Or, related to that critic, there is the *a priori* assertion by critics that metal-free catalysis cannot work, often without even providing a rationale. Working graphene catalysts represent a paradigm change in chemical engineering as they belong to the class of metal-free sustainable catalysts, which are considered a holy grail in catalysis (Boero et al., 2022; Ikeda et al., 2008; Ikeda et al., 2010; Narayanan et al., 2013; Niyogi et al., 2002; Ozaki et al., 2007; Planeix et al., 1994; Qi and Su, 2014; Xiaoyan et al., 2013; Zhang and Dain, 2012; Zhao et al., 2017). Fundamentally, new concepts are apparently always difficult to accept. However, in a very recent study (Seif et al., 2026), we showed that graphene deposited on silicon(111) is reactive to decompose small sulfur-containing compounds. Experimental studies of samples with different grain boundary defect (GBD) densities demonstrated that the reactivity of graphene/Si(111) is defect-driven. In addition, density functional theory (DFT) calculations (Seif et al., 2026) identified the active sites as interfacial defects that form underneath the grain boundary defects, as well as the grain boundaries’ themselves. Thus, the reactivity of graphene/Si(111) is “defect- and support-driven,” perfectly illustrating the title of this volume. The model developed for graphene/Si(111) may also be applicable to other graphene systems. Furthermore, silicon is a metalloid semiconductor, i.e., it is not metal. Therefore, graphene/Si(111) represents a metal-free carbon catalyst that decomposes at least small sulfur-containing compounds. The graphene used here is non-functionalized in the sense that Gr was neither doped nor decorated by adatoms.

## Discussion

**FIGURE 1:**
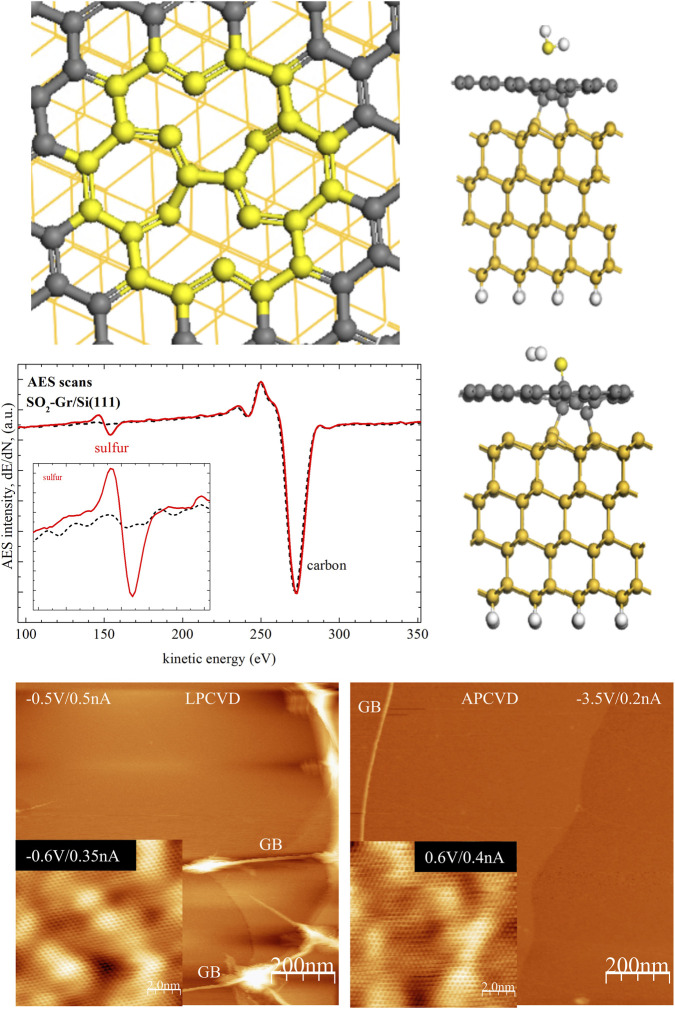
Top left: DFT model of a 5-5-7-7-8-8 grain boundary defect (GBD) in graphene (Gr) (yellow and gray circles) on top of silicon (thin yellow lines). Top right: molecular H_2_S above a GBD (gray) on silicon (yellow circles).Second row, left: Auger electron spectra before (black) and after (red) SO_2_ thermal desorption experiments (similar data were obtained for H_2_S). Second row, right: dissociated H_2_S above a GBD (grey) on silicon (yellow circles). Bottom left column: scanning tunneling microscopy (STM) images of LP Gr (larger GBD density), where GBDs are evident. Bottom right column: STM images of AP Gr (smaller GBD density), where GBDs are mostly absent. The STM insets show atom-resolution scans.

Experimentally, we initially studied Gr/Ru(0001) and used probe molecules such as benzene, alkanes, and water, but all those probes adsorbed only molecularly (Chakradhar and Burghaus, 2014; Chakradhar et al., 2013; Chakradhar et al., 2015; Chakradhar et al., 2016; Sivapragasam et al., 2016a; Sivapragasam et al., 2016b; Sivapragasam et al., 2017). Inspired by studies in liquid–solid organic chemistry and by theoretical work (Borisova et al., 2013; Esrafili et al., 2016; Faye et al., 2016; Komeily-Nia et al., 2020; Long et al., 2011; Mohammadi et al., 2017; Su and Loh, 2013; Widjaja et al., 2017) indicating reactivity toward sulfur compounds, we switched to characterizing SO_2_, H_2_S, and thiophene adsorption on Gr/Ru(0001). Those small sulfur-containing compounds dissociated (Stach et al., 2021a; Stach et al., 2021b; Stach et al., 2023a; Stach et al., 2023b; Stach et al., 2024a). Our first successful example was SO_2_-Gr/Ru(0001); here, SO_2_ does not merely adsorb molecularly but dissociates (Stach et al., 2021a). That system, unfortunately, is not metal-free. Meanwhile, after being joined by a DFT group specializing in incorporating support effects (Ambrosetti and Silvestrelli, 2016; Ambrosetti and Silvestrelli, 2017), a rather simple concept emerged. Most early DFT studies considered only free-standing Gr, which is not very reactive. While Gr supported on reactive substrates becomes reactive, Gr remains inert on less reactive substrates (Carraroa et al., 2018; Cela et al., 2017; Smerieri et al., 2015). The same small sulfur-containing compounds adsorb only molecularly on Gr/SiO_2_ (using the same experimental setup) (Stach et al., 2023b). The idea emerged that if Ru substrates work but SiO_2_ does not, silicon should be tested because it is considered a metalloid with properties intermediate between metals and non-metals (/semiconductors). Initial (pre-screening) DFT calculations on Gr/Si were not encouraging; however, at that time, our DFT studies considered only defect-free Gr/Si. According to DFT, pristine Gr on silicon was not reactive. Another obstacle was that Gr/Si samples are not readily available and are difficult to synthesize. Direct deposition of carbon on Si mainly forms silicon carbides, which exhibit chemistry very different from that of graphene. Fortunately, another group joined the collaboration that specialized in synthesizing Gr on silicon through transfer of Gr initially grown on copper. When Gr is grown on copper by low-pressure chemical vapor deposition (LPCVD synthesis, LP hereafter), samples with rather high densities of grain boundary defects (GBD) are obtained. In contrast, atmospheric-pressure CVD generates only few GBD (APCVD synthesis, AP hereafter). Scanning tunneling microscopy (STM) imaged those GBDs and verified the absence of atomic vacancy defects, large vacancies, and impurities (Figure 1, bottom row). Kinetics experiments combined with spectroscopy (Figure 1, center left) on those Gr/Si(111) samples revealed dissociation of small sulfur-containing compounds. We finally identified a working metal-free graphene catalyst (Seif et al., 2026). This system operates under ultra-high vacuum and involves gas–surface reactions on non-functionalized, clean Gr.

According to DFT, pristine Gr on silicon was not reactive. How, then, can these results be explained? Extensive DFT calculation on different defect structures provided clarification (Seif et al., 2026). The detailed atomic structure of GBDs on Gr/Si is experimentally still unknown. Following essentially a trial-and-error approach, GBDs were eventually modeled in DFT using so-called 5-5-7-7-8-8 defects (see the molecular models in Figure 1, top row and second row right). STM scans (Figure 1, bottom row) imaged those GBDs, which are more frequent for LP GR (Figure 1, bottom row, left) than for AP Gr (Figure 1, bottom row, right). On APCVD Gr, we also obtained similar images at a −0.6 V bias (see inset), although they were somewhat more affected by noise and minor tip instabilities. Most carbon atoms associated with the GBDs remain part of the graphene lattice but become rehybridized, i.e., they appear as Csp (Komeily-Nia et al., 2020) in X-ray photoelectron spectra (XPS) (Seif et al., 2026). Interestingly, according to DFT, C atoms associated with the GBDs can also bound to the Si substrate, i.e., interfacial defects are formed involving C atoms located between the Gr lattice and the Si substrate. A Si-C peak was also observed in XPS scans (Seif et al., 2026). The DFT results suggest that both the GBDs and the interfacial defects are the active sites responsible for decomposition of sulfur-containing compounds (Seif et al., 2026). Experimentally, LP samples, which contain more GBDs, were more reactive than AP samples (Seif et al., 2026). AES measurements (Figure 1, second row left) detected adsorbed sulfur after dosing SO_2_ or H_2_S, thereby revealing dissociation of those molecules.

What is special about sulfur-containing compounds? N_2_O, for example, does not dissociate on Gr/Si (Seif et al., 2026), similarly to ether (CH_3_CH_2_–O–CH_2_CH_3_) (Stach et al., 2025). Sulfur in these compounds possesses empty d-orbitals, which may act as electron acceptors from Gr/Si (Ambrosetti and Silvestrelli, 2016; Stach et al., 2023a; Stach et al., 2023b; Stach et al., 2024a). Those electrons can then transfer into antibonding orbitals of the S-H bonds in the adsorbates, thereby promoting dissociation of the sulfur compounds. Thus, d-electrons are involved (see criticism #1 above). However, the d-electrons are not associated with graphene itself, but rather with the probe molecules (see next paragraph). In contrast, the delocalized electron density, linear structure, and strong N≡N bonding render N_2_O inert on Gr/Si (Seif et al., 2026). For ether, the Si substrate enhances adsorption, but the activation barrier for dissociation remains too high to be thermally overcome, although photocatalytic reactions may still be feasible (Stach et al., 2025).

Any rigorous project should critically re-examine and validate its results. Thus, let us return to the criticisms mentioned in the introduction.

1. Missing d-electrons in Gr: Gr does not possess d-electrons but rather delocalized p-type electrons that give rise to its semi-metallic properties, including high electrical conductivity. It is also true that S-O antibonding orbitals (in the small sulfur-containing compounds used here as probe molecules) are mainly of *p* character, and the contribution of *d* states is probably marginal, although it cannot be completely ruled out. However, e.g., in H_2_S, empty *d*-orbitals can act as electron acceptors from the substrate, thereby promoting H_2_S dissociation (Seif et al., 2026). Regarding the absence of *d*-electrons in graphene, often cited by critics of metal-free catalysis, our point is precisely that *d-*electrons are not a prerequisite for catalytic bond activation. The delocalized *p*-type electrons of graphene, enhanced by interfacial defect states, are sufficient to populate the relevant antibonding states of the adsorbates and thereby drive dissociation.
2. Catalytic vs. stoichiometric behavior: Auger electron spectroscopy (AES), XPS, and Raman demonstrate that Gr is preserved after adsorption of small sulfur-containing compounds (Seif et al., 2026). Thus, the reactions on Ru- and Si-supported Gr are catalytic in nature. Unfortunately, sulfur appears to poison Gr/Si, which prevents sustained formation of a catalytic cycle (adsorption of reactants→desorption of products→…) under vacuum conditions on that system (Seif et al., 2026). Therefore, the catalyst described here would not operate indefinitely. One could imagine, however, removing sulfur under higher-pressure processing conditions. The system studied here is a model system and does not currently have direct industrial application. Nevertheless, Gr-coated Si nanoparticles may represent a commercially interesting material. The Gr layer could suppress oxidation of Si under ambient conditions, which is desirable because SiO_2_ is not reactive (Stach et al., 2023b). Note that Gr/Ru was not poisoned by sulfur (Stach et al., 2021a). Furthermore, a fully closed catalytic cycle is not strictly required for a material to qualify as a catalyst according to the original definition proposed by Berzelius.
3. Intercalation effects: Such effects were apparently observed for sputtered Gr/Ni (Celasco et al., 2016). Although difficult to rule out entirely, intercalation would likely destroy the Gr layer completely, which was not observed for either Gr/Si or Gr/Ru. We did not investigate this systematically; however, CO and CO_2_ did not appear to adsorb on Gr/Si down to approximately 100 K. This observation should largely rule out intercalation and large vacancy defects since CO and CO_2_ would adsorb on exposed Si surfaces.
4. Vacancy defects: Such defects would be observed in STM images (Seif et al., 2026). Some probe molecules, such as N_2_O, which are reactive on bare Si, remained unreactive on Gr/Si, which also argues against the presence of large vacancy defects. If large holes were present in the Gr layer, N_2_O would likely dissociate.

With kinetic experiments described above, one cannot completely rule out extremely small vacancies, such as single atomic vacancies; however, such defects should be visible in STM images (but they were not observed).

Currently, the exact atomic structure of grain boundary defects on Gr/Si(111) remains unknown. The DFT model of GBDs includes relatively large vertices (see Figure 1, top left). However, using nudged elastic band DFT (NEB-DFT), an activation barrier of approximately 1.77 eV (171 kJ/mol) was estimated for the penetration of molecules such as H_2_S through the GBDs. Such a high activation energy makes this process highly unlikely under the experimental conditions.

5. Unidentified impurities/artefacts: Transfer of Gr from copper could potentially transfer trace amounts of copper onto Gr/Si. However, XPS and AES ruled out metallic impurities down to approximately 1% of a monolayer. Atomically resolved STM also ruled out impurities on the atomic scale (Seif et al., 2026). If residual Cu is nevertheless present at extremely low concentrations, it would most likely exist as interfacial impurities, which may themselves be non-reactive. Some amorphous carbon impurities are present due to the air transfer of Gr from copper to Si. Note also that commercial Gr/SiO_2_ samples, which are likewise prepared by transfer of graphene from Cu, were not reactive (Stach et al., 2023b). Theoretically, several possible experimental artifacts exist, including relatively trivial ones, which are discussed in the Supplementary Information of Stach et al. (2023a). Examples include possible effects caused by ion gauge filaments in the vacuum system. Extensive control experiments ruled out such artefacts during studies of Gr/Ru. The same experimental setup was subsequently used for Gr/Si.

## Conclusion and outlook

This is a brief “perspective” article rather than a review. However, we should acknowledge the many other studies devoted to metal-free catalysis—in particular those characterizing carbon catalysts (Narayanan et al., 2013; Niyogi et al., 2002; Planeix et al., 1994; Qi and Su, 2014; Xiaoyan et al., 2013; Zhang and Dain, 2012; Zhao et al., 2017). Several projects have demonstrated that non-functionalized graphene-based materials can exhibit catalytic activity related to heteroatom-induced electronic perturbations, defective carbon sites, or zigzag edges (Boero et al., 2022; Ikeda et al., 2008; Ikeda et al., 2010; Ozaki et al., 2007). Similarly, wrinkles induced by Gr–substrate interactions have been observed to enhance the catalytic properties of graphene (Suna et al., 2023). Numerous studies on (metal)-doped Gr or metal clusters deposited on Gr have also been conducted, although it is perhaps unsurprising that those systems become reactive (see references in Burghaus, 2019)).

As an outlook to our “perspective article,” one may ask: what did we learn from our endeavor, progressing from pure molecular interactions of various molecules to dissociation of sulfur-containing compounds and from ruthenium, copper, and silicon dioxide to silicon substrates? Including extensive literature discussions, we reviewed the various known mechanisms that may render Gr reactive in prior studies (Burghau and s, 2016; Burghaus, 2009; Burghaus, 2014; Burghaus, 2022; Stach et al., 2024b). A fundamentally new aspect of the latest work on Gr/Si(111) arises from a defect–support coupling, where interfacial electronic perturbations activate otherwise inert graphene. Defect rehybridization at grain boundaries, together with interfacial orbital coupling, can enable bond activation in probe molecules. We hypothesize that this model of interfacial defects may be transferable to other graphene–substrate systems, thereby adding a three-dimensional component to the surface science of graphene. Free-standing graphene models may miss key chemistry that emerges only when both defects and supports are explicitly included.

In summary, the concept of noble metal-free catalysis dates back many years (Long et al., 2011; Mohammadi et al., 2017; Qi and Su, 2014; Schreiner, 2003; Su et al., 2010; Wang et al., 2011; Xiaoyan et al., 2013; Zhang et al., 2007), but it has thus far been explored mainly in liquid/solid phase reactions. However, gas–surface reactions are preferred for industrial processes because they allow easier separation and generate less waste (Ertl et al., 1997; Thomas and Thomas, 2005). Are there applications of Gr beyond exploiting its remarkable electronic properties, e.g., in nanoelectronics? There are now sufficient examples demonstrating that Gr can also function as a catalyst (Burghaus, 2022; Seif et al., 2026; Stach et al., 2024b; Suna et al., 2023). Looking forward, our perspective is that graphene has applications as a metal-free catalyst for gas–surface reactions.

## Data Availability

The original contributions presented in the study are included in the article/supplementary material; further inquiries can be directed to the corresponding author.
